# Remote school instruction in Fall 2020 and psychiatric emergencies among adolescents in Los Angeles County

**DOI:** 10.1186/s12888-024-06225-w

**Published:** 2024-11-27

**Authors:** Shutong Huo, Annie Ro, Senxi Du, Andrew Young, Tim A. Bruckner

**Affiliations:** 1 Department of Health, Society, and Behavior, Joe C. Wen School of Population & Public Health, University of California, Irvine, 856 Health Sciences Quad, Irvine, CA 92697-3957 USA; 2 David Geffen School of Medicine, University of California, Los Angeles, USA; 3 Harbor UCLA Medical Center, Torrance, USA; 4 Center for Population, Inequality, and Policy, University of California, Irvine, Irvine, United States

**Keywords:** Adolescent mental health; COVID-19; Psychiatric ED visits

## Abstract

**Objective:**

Schools play an essential role in providing mental health care for adolescents. School closures during COVID-19, as well as re-opening to remote-only instruction in Fall 2020, may indirectly affect the utilization of emergency psychiatric care. We examine COVID-19-related changes in emergency psychiatric care among youth during the school closure and after school reopening (with remote instruction).

**Methods:**

We use Box-Jenkins interrupted time series methods to analyze psychiatric emergency department (ED) visits among patients 10–19 years at LAC + USC Medical Center (LAC + USC) between January 5th, 2018, and December 31st, 2020. We control for the 1st societal shutdown in LA County (i.e., the nine weeks from March 13 to May 14, 2020) when analyzing the potential “return to remote school” shock.

**Results:**

Youth psychiatric ED visits fell by 15.3 per week during the Spring 2020 school closure (*p* < .05). The “return to remote school” coefficient (i.e., August 14th to September 10, 2020), by contrast, is positive but does not reach statistical detection above expected values (*p* = .11). However, the proportion of psychiatric ED visits rises 38% among youth during the “return to remote school” period (*p* = 0.006).

**Conclusion:**

The initiation of Fall 2020 remote instruction corresponded with a greater proportion of youth ED visits that are classified as psychiatric.

## Background

According to the World Health Organization, adolescence is the stage of life between childhood and adulthood, aged 10–19, which represents a key phase of identity formation in which many mental health disorders first present clinically [1, 2]. The National Comorbidity Survey finds that an estimated 49.5% of adolescents in the US have a mental health disorder [3]. Fortunately, schools play an important role in providing mental health services for school-aged adolescents, especially for disadvantaged populations [4, 5]. School health programs serve an important screening, mental health care, and referral function [6]. A recent analysis finds that 13.2% of adolescents (i.e., ~ 3 million) received mental health services from a school setting in the past 12 months [7].

With the onset of the COVID-19 pandemic in the US, most public schools ceased in-person operation in Spring 2020. Public health officials reasoned that ceasing in-person schooling would reduce person-to-person transmission of SARS-CoV-2. Moreover, clinicians and scholars predicted other sequelae, including a “psychiatric pandemic” following school closure, characterized by a rise in symptoms among adolescents [8–10]. These predictions followed two main considerations. First, without in-person instruction, schools may have less capacity to provide these mental health services, which might lead to missed care and exacerbation of conditions, or sub-optimal care in non-routine settings (e.g., the emergency department [ED]). During remote learning, virtual counseling sessions and online mental health resources were not always accessible or effective, particularly for vulnerable students [11]. For example, immigrant families with language barriers and students with disabilities might have had disproportionally fewer opportunities to use online mental health services during COVID-19 (relative to using in-person services pre-pandemic) [12]. Second, social isolation at home (relative to routine social interaction at school) may increase anxiety, fear, boredom, and loneliness—especially among socio-economically vulnerable adolescents [13, 14].

The empirical evidence for this psychiatric pandemic among adolescents, however, is mixed. In the US, overall visits for emergency psychiatric care among adolescents fell in spring 2020 [15–19]. In addition, we know little about whether adolescent psychiatric emergencies responded to the evolving COVID-19 pandemic. In some settings, youth and their parents reported increased emotional, behavioral, and restlessness/inattention difficulties following school closure in Spring 2020 [20]. By contrast, other indicators of mental health generally either remained at pre-pandemic levels (e.g., suicide) or fell (e.g., psychiatric visits to the ED) during Spring 2020 [21]. One study in the US found that admissions to a psychiatric hospital after mid-March in 2020 were reduced compared to the same time period in prior years, but the study used only a small cross-sectional sample [22]. Studies in Italy [23] and Spain [24] also found that adolescent psychiatric ED visits fell after the local COVID-19 lockdown, but they did not control for strong trends pre-COVID-19.

The ongoing social changes as the pandemic progressed suggest that patterns of adolescent psychiatric medical need could change over time. Some studies, for instance, indicated a return to pre-pandemic levels of psychiatric help-seeking for adolescents beginning in late 2020 [18, 19]. In particular, the decision on the part of many school districts to start the Fall 2020 school year remotely could have affected adolescent mental health in unique ways. First, adolescents spending more time at home with parents in Fall 2020 may have led parents to “uncover” existing disorders and refer their children to care. Second, confronting the enduring reality of remote instruction in Fall 2020 among adolescents may have further exacerbated symptoms of disorder above and beyond levels reported in Spring 2020. Third, lack of access to a school health program could lead to failures to detect and treat mental health disorders, thereby leading to more severe symptoms and a rise in help-seeking in non-routine settings, such as the ED. This potential increase in help-seeking in Fall 2020 would differ from the patterns of psychiatric ED utilization in Spring 2020 when the public was more concerned about contracting COVID-19 in hospitals and was discouraged from inundating the ED with non-COVID-19 related concerns.

We know of no previous literature that tests a potential “rebound” in psychiatric ED utilization after school re-openings in Fall 2020. We extend prior work by testing whether, and to what extent, emergency mental health care among adolescents rose above expected values during the first month of Fall 2020 remote instruction. We, unlike earlier work, apply rigorous time-series methods to the investigation. We focus on Los Angeles, the most populous county in the US and the second-largest public school system in the county. The Los Angeles Unified School District (LAUSD) operated the entire academic year of 2020–21 with remote instruction, affecting 664,774 students [25, 26].

## Methods

### Data and variables

We analyzed psychiatric ED visits among patients 10–19 years in the LAC + USC Medical Center (LAC + USC) between January 5th, 2018, and December 31st, 2020. Our data come from the LAC + USC Medical Center’s Vizient Health System Data, a hospital billing and administrative claims database that records all medical center patient visits. LAC + USC Medical Center ranks among the busiest hospitals in the US in terms of the volume of psychiatric ED visits and ED visits overall. The majority of patients who seek care at LAC + USC Medical Center qualify for publicly-funded health insurance (e.g., Medi-Cal, California’s version of the Federal Medicaid program). All project activities were reviewed and approved by the USC Institutional Review Board (HS-19–00890), which served as a reliance for the UC Irvine Institutional Review Board.

Consistent with the federal government’s classification scheme, we identified a psychiatric ED visit using Clinical Classification Software (CCS), which combines individual ICD-9 diagnoses into clinically meaningful categories. We defined a mental health-related diagnosis with any ICD-9 code ranging from 650 to 670 in the CCS, [27] consistent with the definition used by the Healthcare Cost and Utilization Project (HCUP) and other federal databases. This federally endorsed classification scheme allows direct comparison of our results to other work [28]. These CCS codes include but are not limited to the following conditions: mood, anxiety, schizophrenia, and behavioral disorders; suicide attempts; self-harm; and alcohol and substance-related disorders [27]. The complete list of CCS codes is available in the online supplement.

We used the count of psychiatric ED visits as our key outcome variable and aggregated the number of visits into 7-day periods. Proportion of psychiatric ED visits is a secondary dependent variable. Proportion, unlike raw counts, may capture a rise in proportion of adolescent ED visits that are psychiatric (vs. non-psychiatric) that coincides with the beginning of the remote school year. We calculated the proportion of psychiatric ED visits due to mental health/substance use disorders among adolescents by dividing this value by the *total* number of youth ED visits each week.

The key independent variable was the school reopening in August 2020. LA schools differed in their start date for online instruction in the 2020–21 school year. Start dates ranged from Aug 12 to 27, 2020. Therefore, we coded the four weeks from August 14 to September 10 that we identified as start of the school year in Fall 2020 as “1”. We coded all other weeks as “0.”

The first societal shutdown in LA County (i.e., the nine weeks from March 13 to May 14, 2020) may have reduced help-seeking in the ED for all types of care, including psychiatric care. To control for this sudden decline in ED visits, we adjusted for this “shutdown” effect when examining the independent association between the beginning of the remote school year in Fall 2020 and psychiatric ED visits. To do so, we aggregated ED counts such that each 7-day period started on Friday and ended on Thursday. The Friday, 3/13/20, the date that the Trump Administration declared a national emergency due to COVID-19, serves as the first day of the exposed “anchor” 7-day period. In total, we examined 156 full weeks of ED visits beginning January 5, 2018, and ending December 31, 2020. The USC Institutional Review Board (HS-19–00890), which serves as a reliance for the UC Irvine Institutional Review Board, approved all project activities. All data were de-identified to conform to Health Insurance Portability and Accountability Act requirements.

### Analysis

We use psychiatric ED visits as an important gauge of help-seeking in that it may indicate either a lack of outpatient mental health care options, a rise in prevalence of disorder, or both. If we observe a rise in the number of youth psychiatric ED visits after schools reopen, it would cohere with a “psychiatric pandemic” induced by societal disruption and uncertainty. If instead the proportion of psychiatric ED visits rebound to the pre-pandemic level, it would suggest that mental health issues among youth featured more prominently in Fall 2020 relative to non-psychiatric conditions.

Psychiatric ED visits exhibit well-characterized temporal patterns, including seasonality, trend, and the tendency for high or low values to be “remembered” into subsequent months. These patterns, referred to as autocorrelation, complicate classical tests of association because the expected value of a patterned series is not its mean. To address such autocorrelation, we employed autoregressive, integrated, moving average routines (ARIMA) recommended in the literature [29, 30]. These routines, devised by Box and Jenkins, identify and remove any autocorrelation in the dependent variable series. These routines express autocorrelation as a product of “autoregressive” (AR), “integrated” (I), and “moving average” (MA) parameters, collectively referred to as ARIMA models. The residuals of these ARIMA models meet the assumptions of correlational tests in that they have an expected value of 0 and exhibit no serial dependence. After removing autocorrelation, the analyst inserts the “interruption” exposure variable to determine whether the dependent variable's residual values move away from their expected value during the hypothesized interruption.

We implemented the above time-series approach on both count and proportion of psychiatric ED visits with the following steps. First, we used the time-series autocorrelation and partial autocorrelation functions to identify potential AR, I, or MA parameters for the 114 weeks of psychiatric ED visits, which covers the pre-pandemic period in LA County (i.e., Jan 5, 2018, to March 12, 2020). Second, we added ARIMA parameters to express autocorrelation identified in its residual values (i.e., error term) in the first 114 weeks. Third, we estimated the model formed by adding the “school reopen” binary variable in the model from step 2 and controlling for the first stay-at-home order restriction in Spring 2020. We hypothesize a synchronous relation (i.e., psychiatric ED visits among adolescents rise during the beginning of Fall instruction) and, unlike step 1, used all 156 weeks of the entire series (i.e., to Dec 31, 2020). Fourth, we inspected the residuals of the time-series equation to ensure that they exhibited no autocorrelation. We performed all time-series analyses using Scientific Computing Associates (River Forest, IL).

## Results

Table 1 provides the sociodemographic characteristics of the adolescent population that presented to the clinic over the test period. During the test period (from January 5th, 2018, to December 31st**, 2020**), 4784 patients visited LAC + USC (accounting for 6022 visits) for psychiatric emergencies among age group 10–19 years. More females than males have psychiatric emergencies. About two thirds of the patients report Hispanic origin and over two thirds of the patients are covered by Medicaid.

**Table 1:** Demographic information of patients with psychiatric emergencies in Los Angeles County and USC Hospital among the age group 10–19 years

	Before lockdown (week 1 to week 114)	During school closure (week 115 to week 136)	After remote school year begin (week 137 to week 156)	Total psychiatric emergencies (week 1 to week 156)	Total emergencies (week 1 to week 156)
N	4803	528	691	6022	28,845
Age (mean)	15.03	15.91	15.61	15.17	15.43
Sex (male, %)	46.11	50.48	47.26	46.58	50.33
Hispanic Origin (%)	67.12	59.47	62.66	65.95	78.12
Medicaid (%)	70.08	62.69	66.86	69.06	76.91

During school closure in 2020, relatively fewer patients are female, Hispanic, and with Medicaid insurance compared with the pre-pandemic period. After remote school beginning in 2020, the proportion of female, Hispanic, and Medicaid insured patients with psychiatric emergencies rose.

**Fig. 1:**
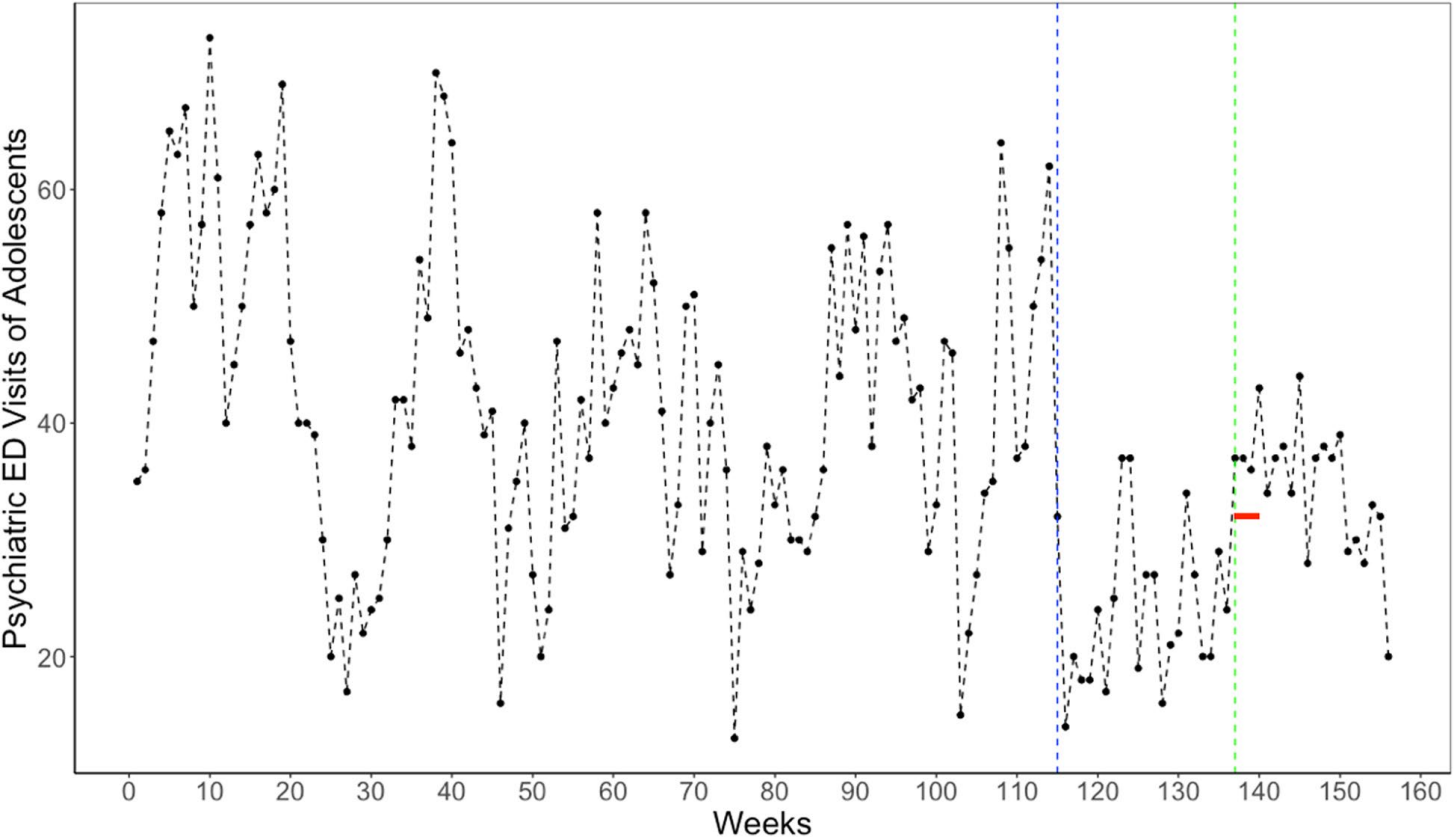
Counts of Psychiatric Emergencies among youth in LAC + USC over 156 weeks (Jan. 2018-Dec.2020). Notes: The figure shows the counts of Psychiatric Emergencies among Youth aged 10–19 years in Los Angeles County and USC Hospital over 156 weeks from Jan 5, 2018 to Dec 31, 2020. The red horizontal line indicates August 14 through September 10, 2020. The blue and green vertical lines indicate the week 115, the first week of 1st societal shutdown, and week 137, the first week of school reopening in 2020

Figure 1 plots the weekly count of psychiatric ED visits among adolescents (mean = 38.60; standard deviation [SD] = 13.60) over the 156 weeks spanning Jan 5, 2018, to Dec 31, 2020. The series exhibits considerable variation (weekly range:13 to 73). The crude plot appears to show a reduction in visits during the 1st set of societal restrictions and a modest rebound during school reopening in August 2020.

Using the autocorrelation and partial autocorrelation functions of the series before societal restrictions (i.e., weeks 1 to 114) to diagnose temporal patterns, we identified patterns such that psychiatric ED visits were “remembered” one week later, albeit in diminishing amounts. We also detected monthly sequences occurring every 4th week. After removal of this autocorrelation (i.e., AR1 and MA4 parameters) from a time series, the weekly mean is zero and values are serially independent of one another.

Table 2 summarizes the time-series model in which we examined the return to remote Fall 2020 instruction. The “return to school” coefficient for this time frame (i.e., August 14th to September 10, 2020) is positive, but does not reach conventional levels of statistical detection (coef = 10.27, SE = 6.56, *p* = 0.12). The shutdown coefficient, however, indicates a substantial reduction in psychiatric ED visits during the Spring 2020 shutdown (coef = -15.33, SE = 6.37, *p* < 0.05).

**Table 2:** Time Series Results of the Count of Psychiatric Emergencies in Los Angeles County and USC Hospital among the age group 10–19 over 156 weeks from Jan 5, 2018, to Dec 31, 2020, as a function of COVID-19 lockdown, return to Fall School, and autocorrelation

	Coefficient	SE
Constant	39.140	2.642***
Lockdown period, March 13 to May 8, 2020	-15.335	6.367*
Begin remote school year, Fall 2020	10.273	6.557
ARIMA parameters		
AR1	0.655	0.063***
MA4	-0.184	0.082*

^***^p < .001, **p < .01, *p < .05, two-tailed

**Fig. 2:**
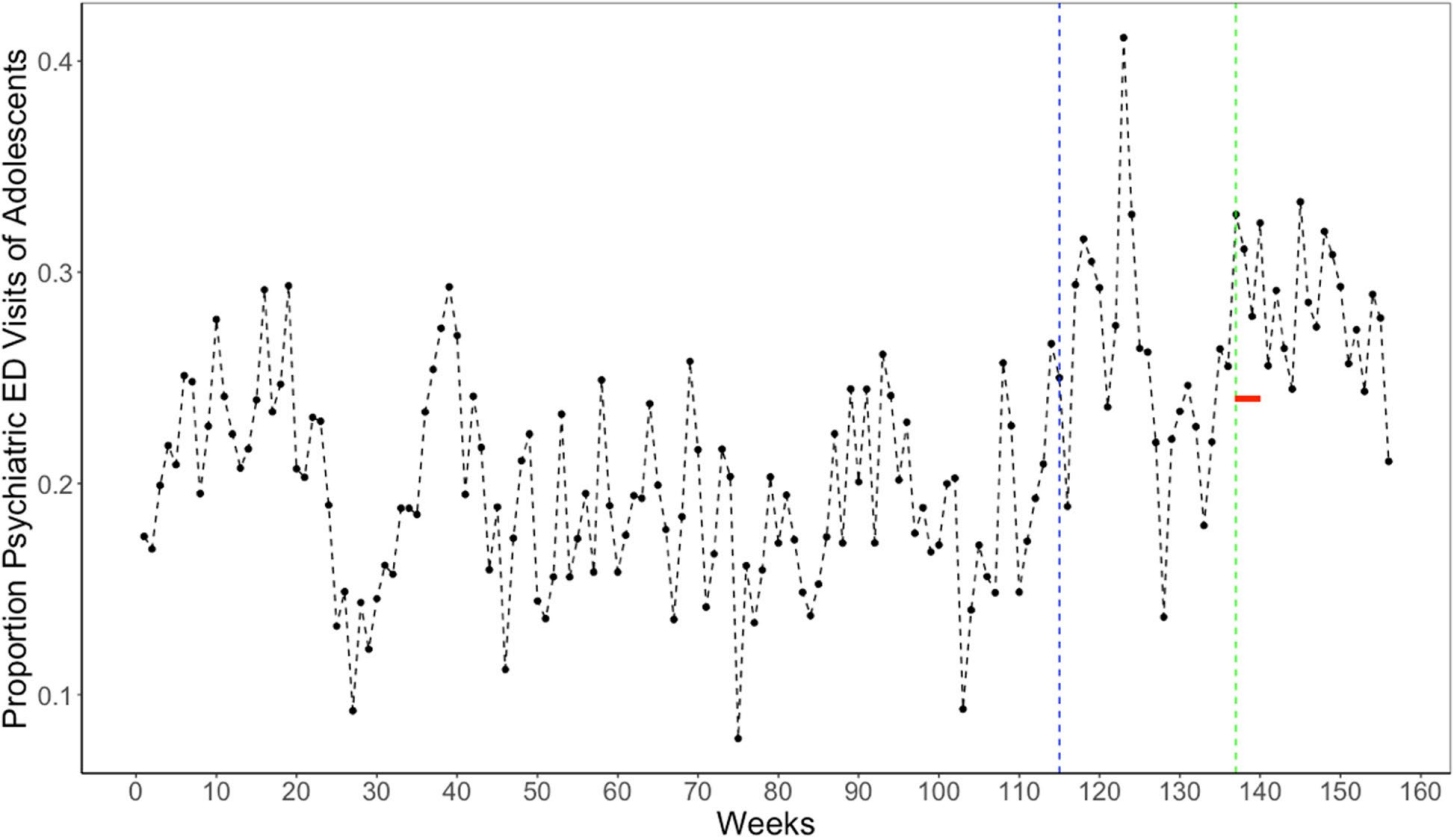
Weekly proportion of Psychiatric Emergencies among youth in LAC + USC over 156 weeks (Jan. 2018-Dec.2020). Notes: The figure shows the weekly Proportion of Psychiatric Emergencies among Youth aged 10–19 years in Los Angeles County and USC Hospital over 156 weeks from Jan 5, 2018, to Dec 31, 2020. The red horizontal line indicates August 14 through September 10, 2020. The blue and green vertical lines indicate the week 115, the first week of 1st societal shutdown, and week 137, the first week of school reopening in 2020

We then examined the possibility that the weekly *proportion* of ED visits that are considered psychiatric among adolescents rose during the “return to school” period in Fall 2020. Figure 2 plots this proportion over the test period. The mean proportion over 156 weeks is 0.214 (SD = 0.056; range = 0.079 to 0.411). The crude plot suggests a “rebound” during school reopening in August 2020. The peak of the plot during summer 2020 is the week of societal reopening. In the Table 3, using the autocorrelation and partial autocorrelation functions of the series before societal restrictions (i.e., weeks 1 to 114), we found AR1 and MA6 parameters. After including these parameters in the time-series equation, the interrupted time series results indicate a statistically detectable rise in the proportion of psychiatric ED visits among adolescents during the “return to school” period (coef = 0.079, SE = 0.029. *p* = 0.006). This rise equates to a 38.1% increase above the base rate that is statistically attributable to the return to remote School instruction in Fall 2020.

**Table 3:** Time Series Results of the Proportion of Psychiatric Emergencies in Los Angeles County and USC Hospital among the age group 10–19 over 156 weeks from Jan 5, 2018, to Dec 31, 2020, as a function of COVID-19 lockdown, return to Fall School, and autocorrelation

	Coefficient	SE
Constant	0.210	0.009***
Lockdown period, March 13 to May 8, 2020	0.060	0.025*
Begin remote school year, Fall 2020	0.080	0.029**
ARIMA parameters		
AR1	0.557	0.069***
MA6	-0.186	0.081*

^***^p < .001, **p < .01, *p < .05, two-tailed

## Discussion

The study examined whether COVID-19 related closures of in-person school instruction in 2020 affected adolescent mental health. We used the count of psychiatric ED visits among youth, as well as the proportion of ED visits classified as psychiatric, as population indicators of adolescent mental health. Results from one of the largest hospitals in the US show a significant and acute reduction in psychiatric ED visits after the first stay-at-home order. Then, after school reopened to remote-instruction only in August 2020, counts of adolescent psychiatric ED visits rebounded but did not rise above pre-pandemic levels. The *proportion* of adolescent psychiatric emergencies, by contrast, rose substantially in Fall 2020 after the start of remote-instruction. Return to remote-instruction appears to have increased help-seeking for mental health disorders (relative to help-seeking for non-mental health reasons) among youth precisely at a point where school-based mental health resources were limited.

A known pattern of seasonality in child and adolescent psychiatric inpatient admissions closely follows the school year, with the most admissions occurring during months of academic instruction, and the fewest occurring during summer months as well as weeks of winter holidays [31]. These seasonal trends have been attributed to increased recognition of mental health disorders by teachers or school counselors who may be more likely to refer students for treatment than parents, academic stressors, and negative social interactions at school, including bullying [31]. Our methods, however, control for this predictable seasonality. Therefore, the unexpected rise in proportion of psychiatric emergencies during Fall 2020 in Los Angeles cannot be attributed to seasonality.

At the start of school reopening to remote instruction, parents may more directly observe that their children struggle with access to technology, shifts in routine, and social isolation [32]. A rise in proportion therefore may reflect parents’ increased willingness to uncover mental disorders among their children and seek emergency care (relative to seeking emergency care for non-psychiatric reasons). Schools engaged in remote learning have recently considered how to foster school connectedness as a means of supporting youth mental health [33]. Such efforts may include targeting mental health and wellbeing within the remote instruction setting. In addition, the increasing availability of telehealth services, in response to the COVID-19 pandemic, may help students cope with mental disorders [34]. During the pandemic, Medi-Cal expanded its coverage for telehealth services, which may have provided an alternative to in-person psychiatric care [35]. Although tele-mental health services are underutilized by Latinx populations and those without private insurance, their increased availability during this period may have contributed to a reduction in the need for ED visits [36].

There are many factors unique to the patient population served by LAC + USC that may contribute to lack of youth access to routine care. LAC + USC primarily serves Hispanic/Latino patients and Medicaid-insured patients. In the US, Hispanic/Latino adolescents had lower odds of receiving adequate mental health care when compared to non-Hispanic white adolescents [37]. In addition, school closure and home confinement may most acutely affect minority students and those who are socially and economically disadvantaged (including the homeless and undocumented immigrants) [38]. School-based mental health services could provide their key avenue to care. After the school reopened, these students may have been more likely to be referred to psychiatric care by teachers and nurses.

Our analysis makes several key contributions. First, unlike earlier work, we use rigorous time-series methods to control for the strong rival of autocorrelation. Second, we formally test the possibility of a rebound in psychiatric ED visits after school reopened in LASUD. Third, we examine a well-delineated start of “stay-at-home” orders in LA county and of “return to school” and permit a straightforward test of help-seeking changes. Thirdly, our findings align with national trends which show a decrease in psychiatric ED visits among children and adolescents during the pandemic [18, 39].

Limitations include that we did not have full information on the entire adolescent population in LA County. This circumstance leaves open the possibility that adolescents who ordinarily seek care in our study hospital (LAC + USC Medical Center) sought care elsewhere during the pandemic and remote school reopening. We view this possibility as unlikely for two reasons. First, the decision of where youth seek ED care (as opposed to routine non-emergency care) largely involves location—that is, persons typically go to the closest hospital [40]. We know of no evidence that indicates large shifts in residence of youths—within Los Angeles—that would have led to preferentially seeking ED care elsewhere only in Spring 2020 but not in Fall 2020. Second, we assessed quarterly data on all youth ED visits on two adjacent LA hospitals for the years 2019 and 2020. None reported a specific compensatory pattern of *increases* in adolescent help-seeking during Spring 2020, followed by a *decrease* in Fall 2020. The patterns of visits, instead, looked similar to the pattern for ED care at LAC + USC.

We also, owing to small counts of weekly ED visits by psychiatric subtype, could not examine which types of mental health disorders accounts for a rebound in the proportion of psychiatric ED visits when Fall 2020 remote instruction began. ED visits also represent an extreme indicator of mental health disorders. We, however, know of no other weekly measure of adolescent mental health in LA or elsewhere in the US. The development of such a surveillance system would be important for future understanding adolescent mental health patterns. Owing to the aggregate-nature of our data, we could not examine whether psychiatric emergencies rose among adolescents who had a caregiver suffer from COVID-19 infection. We, however, view this rival explanation for our findings as unlikely given the relatively low morbidity burden of COVID-19 in August 2020 in Southern California [41]. This explanation may take on more salience as the pandemic matured in Winter 2020/21; we await future work using 2021 data to address this question.

Results in our large LA County hospital do not suggest a “psychiatric pandemic” of ED visits among adolescents in 2020. We, however, encourage replication efforts in other regions, using both ED and other clinical data, before making population-level conclusions. In addition, future work may identify at-risk youth subgroups with unmet psychiatric needs—especially when school mental health programs may have been interrupted.

## Conclusions

In conclusion, this study demonstrates the impact of COVID-19 school closures on adolescent mental health. The findings reveal a significant decrease in psychiatric ED visits during stay-at-home orders, followed by a rebound when schools shifted to remote instruction. The proportion of psychiatric emergencies among adolescents also increased during this period. These results highlight the importance of schools in supporting student mental well-being and emphasize the need for effective interventions during educational disruptions. This study provides valuable insights for policymakers, educators, and healthcare professionals to address the mental health needs of adolescents during school closures.

## Data Availability

The data that support the findings of this study are available from LAC + USC Medical Center but restrictions apply to the availability of these data, which were used under license for the current study, and so are not publicly available. Data are however available from the authors Dr.Annie Ro upon reasonable request and with permission of LAC + USC Medical Center.

## Abbreviations

ED: Emergency department
LAUSD: The Los Angeles Unified School District
LAC + USC: Los Angeles County + University of Southern California Medical Center
CCS: The Clinical Classification Software
HCUP: The Healthcare Cost and Utilization Project
ARIMA: Autoregressive, integrated, moving average
